# Study on Identification Method of Pulmonary Nodules: Improved Random Walk Pulmonary Parenchyma Segmentation and Fusion Multi-Feature VGG16 Nodule Classification

**DOI:** 10.3389/fonc.2022.822827

**Published:** 2022-03-16

**Authors:** Yanrong Zhang, Lingyue Meng

**Affiliations:** Heilongjiang Key Laboratory of Electronic Commerce and Information Processing, Computer and Information Engineering College, Harbin University of Commerce, Harbin, China

**Keywords:** CT image, lung parenchyma segmentation, random walk, volume local direction ternary pattern, Texture features, gray features, VGG16

## Abstract

**Purpose:**

The purpose of this study was to realize automatic segmentation of lung parenchyma based on random walk algorithm to ensure the accuracy of lung parenchyma segmentation. The explicable features of pulmonary nodules were added into VGG16 neural network to improve the classification accuracy of pulmonary nodules.

**Materials and Methods:**

LIDC-IDRI, a public dataset containing lung Computed Tomography images/pulmonary nodules, was used as experimental data. In lung parenchyma segmentation, the maximum Between-Class Variance method (OTSU), corrosion and expansion methods were used to automatically obtain the foreground and background seed points of random walk algorithm in lung parenchyma region. The shortest distance between point sets was added as one of the criteria of prospect probability in the calculation of random walk weight function to achieve accurate segmentation of pulmonary parenchyma. According to the location of the nodules marked by the doctor, the nodules were extracted. The texture features and grayscale features were extracted by Volume Local Direction Ternary Pattern (VLDTP) method and gray histogram. The explicable features were input into VGG16 network in series mode and fused with depth features to achieve accurate classification of nodules. Intersection of Union (IOU) and false positive rate (FPR) were used to measure the segmentation results. Accuracy, Sensitivity, Specificity, Accuracy and F1 score were used to evaluate the results of nodule classification.

**Results:**

The automatic random walk algorithm is effective in lung parenchyma segmentation, and its segmentation efficiency is improved obviously. In VGG16 network, the accuracy of nodular classification is 0.045 higher than that of single depth feature classification.

**Conclusion:**

The method proposed in this paper can effectively and accurately achieve automatic segmentation of lung parenchyma. In addition, the fusion of multi-feature VGG16 network is effective in the classification of pulmonary nodules, which can improve the accuracy of nodular classification.

## 1 Introduction

According to the World Health Organization in 2019, cancer is the leading cause of death in most countries (1). Following up on 2018, the International Agency for Research on Cancer (IARC) team published its latest global cancer statistics report, in January 2021, in the Journal of Cancer Clinicians, a respected journal of the American Cancer Society. The report estimates global incidence and deaths from major types of cancer in 2020. Among them, the incidence of lung cancer in the male and female population accounted for 11.4%, ranking the second, the number of deaths accounted for 18.0%, ranking the first (2).

The cure of lung cancer depends on the detection of the disease at the initial stage, and effective diagnostic methods can lead to a reduction in the incidence of lung cancer (3). At present, Computed Tomography (CT) has become the most widely used imaging method in clinical screening of lung cancer, with the advantages of fast scanning speed and high image resolution (4). According to The National Lung Screening Trial (NLST), compared with conventional radiography, low-dose CT screening can detect tumors at an early stage of cancer, reducing the mortality rate by 20.0% and increasing the positive screening rate by more than three times (5). However, due to the excessive number of whole lung CT images, the labeling accuracy of pulmonary nodules is only 74.9% in the case of full manual scanning (6). A large number of investigations have found that computer-aided design systems have the potential to improve the sensitivity, specificity, accuracy, and cost-effectiveness of lung cancer screening programs.

At present, computer-aided diagnosis system is divided into two categories: computer diagnosis system based on machine learning and computer diagnosis system based on deep learning. Among them, the computer diagnosis system based on machine learning has the following implementation contents: (1) image preprocessing; (2) Segmentation and extraction of target region; (3) feature extraction; (4) Classification and recognition (7).Computer diagnostic systems based on deep learning are mainly Convolutional Neural Networks (CNN) and its improved networks, such as VGG16, AlexNet, ResNet, etc., which can extract high-dimensional features from lung CT images through its own network structure, and then realize the classification of pulmonary nodules.

Accurate segmentation of pulmonary parenchyma is a key step in the diagnosis of pulmonary nodules, and reliable imaging features can improve the accuracy of the diagnosis of pulmonary nodules (8). In medical diagnosis based on traditional machine learning methods, the random walk algorithm of manual interaction is recognized in the segmentation process, and texture features are also input into the classifier as an important factor (9–14). In medical diagnosis research based on deep learning, scholars directly take medical images as the input of the network and complete diagnosis according to the structure of the network itself (15–17). Although the random walk of artificial interaction has achieved a good effect in the segmentation of lung parenchyma, the segmentation efficiency is not high due to the influence of human selection to a large extent. In feature selection, although a few scholars input depth features into traditional classifiers or interpretable features into neural networks, the nodular features input into neural networks are two-dimensional features, while the single depth features lack interpretability (18, 19)..

Therefore, in order to improve the efficiency and accuracy of lung parenchyma segmentation, the random walk segmentation algorithm based on manual interaction was modified into an automatic segmentation algorithm, and the segmentation accuracy was improved by adding the minimum distance index between sets. At the same time, in order to improve the accuracy of nodular classification and determine the influence of different features on nodular classification, we proposed to extract gray scale features and texture features of the region of interest using gray histogram and Volume local direction ternary pattern (VLDTP) method respectively. Different from other texture features, our texture feature method is to integrate the idea of three-dimensional gray co-occurrence matrix into the local three-valued model, so as to obtain the spatial texture features of pulmonary nodules. Then, the interpretable grayscale features and texture features of pulmonary nodules were input into the trained VGG16 network and fused with the nodular depth features in series mode. Finally, pulmonary nodules classification was completed, in which the weight parameters of VGG16 network model were obtained by ImageNet data set training. Experiments show that the proposed random walk automatic segmentation algorithm can avoid the influence of human factors and get better segmentation results than the original method. At the same time, the input of gray scale feature and volume local direction ternary pattern texture feature in VGG16 network is more helpful to the medical diagnosis of pulmonary nodules.

## 2 Materials and Methods

**Figure 1:**
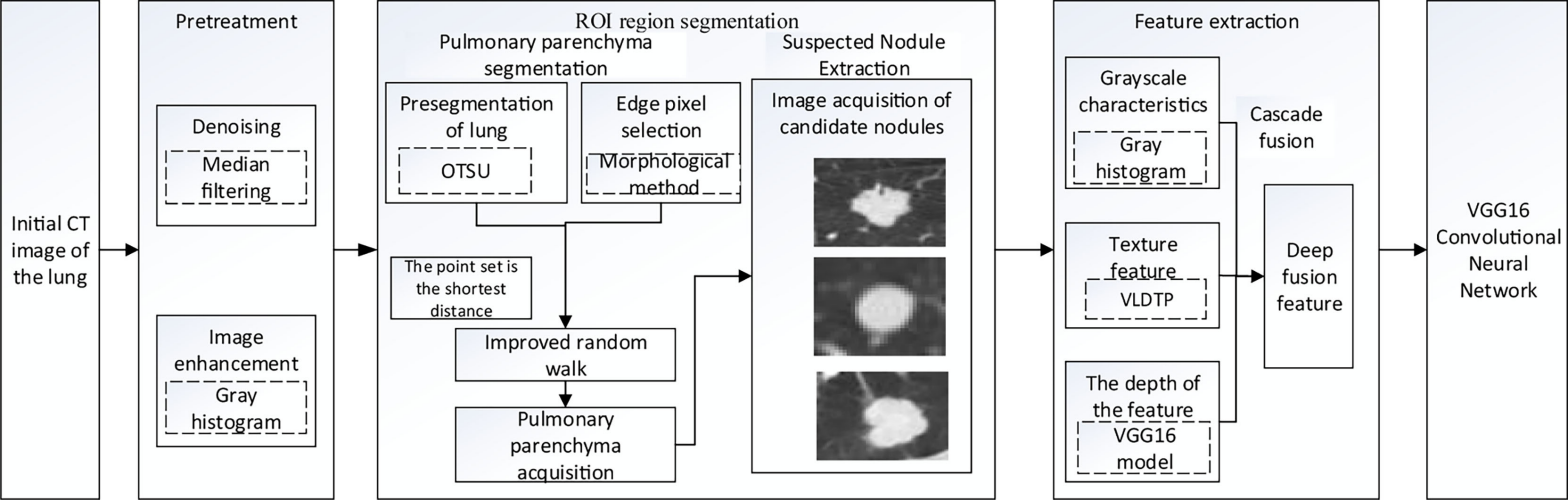
Overall roadmap of the experiment.

Our study used anonymized data extracted from public databases. **Figure 1** shows the specific experimental flow.

### 2.1 Dataset

This study was conducted using The Lung Image Database Consortium (LDC-IDRI) collected by the National Cancer Institute (NCI), which contains chest medical image files (CT, computed tomography; DX, Digital Radiography;(CR, computed Radiography) and corresponding lesion diagnosis labeling file (XML file). It also has some auxiliary metadata information, including patient ID, image location, section distance, resolution, etc. Each subject had an XML file of two stages of a doctor’s diagnosis, In the first stage, four physicians independently diagnosed and labeled nodules and non-nodules. In the second stage, doctors reviewed the diagnosis results of the other three doctors to determine the final diagnosis result of pulmonary nodules. **Table 1** shows a summary of the dataset.

**Table 1:** Summary of dataset.

Dataset	The number of subjects was selected for this study	The amount of experimental data was selected in this study	Selection criteria	Other
LIDC-IDRI	119	1000	Get the images in order of the folders in the dataset.	We extracted nodules based on the location of nodules marked by the doctor in the XML file.

**Figure 2:**
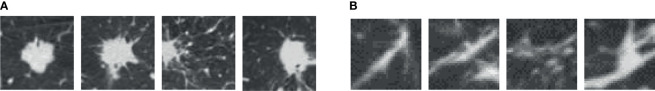
Nodules and suspected nodules in pulmonary CT images; **(A)** Nodules; **(B)** Suspected nodule.

Since the unprocessed CT image contains noise and has low resolution, median filtering is used to de-noise the data, and then the CT image is normalized to a uniform size. The region of interest is mainly extracted from the nodules and suspected nodules marked by doctors. **Figure 2** shows the extracted nodule and the suspected nodule area. In our experiment, 1000 nodules and suspected nodules were selected from the files of 119 patients in sequence as experimental data, including 500 nodules and suspected nodules respectively, which were divided into training set and test set in the ratio of 8:2.

### 2.2 Automatic Segmentation of Lung Parenchyma in CT Images Based on Random Walk Algorithm

**Figure 3:**
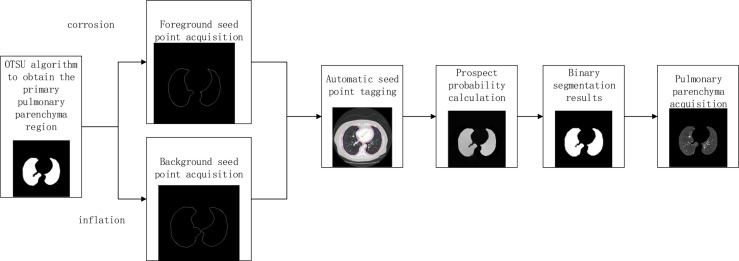
Flowchart of lung parenchyma segmentation based on automatic random walk algorithm.

L. Grady published the article “Random Walks for Image Segmentation” in the journal On Pattern Analysis and Machine Intelligence in 2006. In this paper, the random walk algorithm is applied to image segmentation for the first time (20). Ozen Y. and Kose C (21). pointed out that when the random walk algorithm was used for artificial interactive lung parenchymal segmentation, firstly, the seed points should be marked. The blue foreground seed points were placed in the two lung lobes, and the red background seed points were placed above the chest cavity. Secondly, the user-specified seed point is used as the initial value of the seed. Calculate the probability of arriving at the seed point for the first time in the process of random walk, and mark the first seed point that the unlabeled pixel is most likely to reach as the pixel with the same marked value according to the probability size. Finally, according to the labeling results, the images were divided into foreground and background regions to achieve segmentation of lung parenchyma. In order to reduce the randomness of seed point selection and improve the efficiency of lung parenchyma segmentation, the manual interactive seed point selection was planned to be replaced by computer automatic seed point selection in this paper because of the randomness and long time consumption of manual seed point selection. The specific implementation process is shown in **Figure 3**.

#### 2.2.1 Automatic Selection of Seed Points

In order to improve the working efficiency of random walk segmentation of lung parenchyma, this paper plans to replace the manual interactive seed point selection method with the automatic computer automatic seed point selection method. The specific methods are as follows: Preliminary segmentation of lung parenchyma was performed using OTSU algorithm (22). The expansion boundary points and corrosion boundary points of the initial segmentation results were obtained by mathematical morphology method (23), and the set of two boundary points was used as the background and foreground seed points of the random walk algorithm, so as to realize the automatic acquisition of seed points.

#### 2.2.2 Calculation of Weight Function

Due to the existence of unclear segmentation boundary in the acquisition of automatic seed points, the closer the distance between the pixel vertex and the target seed point is, the greater the enhancement amplitude is. Therefore, in this paper, the shortest distance variable of the set is introduced into the weight function calculation of the random walk algorithm. All pixel vertices except seed points in the graph are included in set *V_U_* = (*v_1_*, *v_2_*, , *v_i_*), The coordinate of a pixel point is $v_{i}(x_{v_{i}},y_{v_{i}})$ , and the seed point is included in set *V_M_* The minimum distance between each pixel point *v_i_* and the set *V_M_* was calculated, and the minimum distance variable was used as one of the measurement criteria for segmentation of the target region, so as to avoid missegmentation caused by image gray information and improve the segmentation accuracy of lung parenchyma.

##### 2.2.2.1 Definition of the Shortest Distance Between Each Pixel Point and the Seed Point


(1) $$d(v_{i},Z)=\underset{z_{j}\in Z}{\text{min}}\left||v_{i}-V_{M}|\right|=\underset{m_{j}\in V_{M}}{\min}\sqrt{{\left(x_{v_{i}}-x_{m_{j}}\right)}^{2}+{\left(y_{v_{i}}-y_{m_{j}}\right)}^{2}}$$

(2) $$V_{M}=F\cup B,V_{M}⊄V_{U},F\cap B=\phi$$

Where all pixel vertices are contained in set *V* = (*v_1_*, *v_2_*, , *v_i_*),The coordinate $v_{i}(x_{v_{i}},y_{v_{i}})$ of a pixel, *V_M_* represents the set of all seed points, Coordinate $m_{j}(x_{m_{j}},y_{m_{j}})$ of a seed point in *V_M_*, *F* represents the set of foreground seed points, *B* represents a set of background seed points.

##### 2.2.2.2 Definition of the Shortest Spatial Distance Similarity of the Shortest Edge Between any Pixel Point and Every Foreground Seed Point and Background Seed Point in the Image


(3) $$G_{v_{m}v_{i}}=\exp\left[-\partial .\frac{d(F,v_{i})+d(B,v_{i})}{\underset{f\in F}{\max}\left\{d\left(f,B\right)\right\}}\right]$$

Where, $\underset{f\in F}{\max}\left\{d\left(f,B\right)\right\}=\underset{f\in F}{\max}\left(\underset{b\in B}{\min}\left||f_{j}-b|\right|\right)$ represents the maximum distance of all distances from a point in the foreground seed point set to the nearest point in the background seed point set. $d\left(F,v_{i}\right)=\underset{f\in F}{\min}\left||v_{i}-f|\right|$ represents the shortest distance from pixel point *v_i_* to the set of foreground seed points, and $d(B,v_{i})=\underset{b\in B}{\min}\left||v_{i}-b|\right|$ represents the shortest distance from pixel point *v_i_* to the set of background seed points. The value of ∂ is 0.048

##### 2.2.2.3 Random Walk Boundary Weight Calculation


(4) $$W_{ij}=\exp\left[-\left[\beta_{1}\sqrt{{\left(g_{i}-g_{j}\right)}^{2}}+\beta_{2}\overset{n}{\underset{z=1}{\min}}G_{v_{z}v_{i}}\right]\right]$$

Where, *g_i_* is the gray value of vertex *v_i_*, *β*_1_ is the weight of pixel gray feature, with a value of 90, and *β*_2_ is the weight of the feature of the shortest distance between pixel and seed point set, with a value of -51.5.

##### 2.2.2.4 Construction of Laplace Matrix

The probability of each vertex moving to the labeled seed point can be solved by solving the positive definite linear equation of the Laplace matrix. Construct the Laplace matrix of Figure *G*= (*V, E*):


(5) $$L_{ij}=\left\{ \begin{array}{l} d_{i},\text{ i}=\text{j}\\ -w_{ij},{\text{ is adjacent to v}}_{j}\\ 0,\text{ other} \end{array}\right.$$

Let’s define a *m* × *n* dimensional diagonal matrix *C*, and define a Laplace operator:


(6) $$L=A^{T}CA$$

*A* is the edge-vertex association matrix of *m* × *n*, which includes every vertex *v_i_* and edge *e_ij_* on the graph.


(7) $$A_{e_{ij}v_{k}}=\left\{ \begin{array}{l} +1,\text{ if i}=\text{k}\\ -1,\text{ if j}=\text{k}\\ 0,\text{ other} \end{array}\right.$$

##### 2.2.2.5 Solving Dirichlet Problem

The key of the random walk algorithm is to convert the vertex first arrival probability into Dirichlet problem. The Dirichlet integral can be converted into the following form:


(8) $$D[x]=\frac{1}{2}{\left(Ax\right)}^{T}C(Ax)=\frac{1}{2}\sum_{e_{ij\in E}}w_{ij}{\left(x_{i}-x_{j}\right)}^{2}$$

Where *x* is the probability of pixel points, *L* is the semi-positive definite combined Laplace matrix, so the only solution of *D*[*x*] is the minimum solution, namely the discrete harmonic function.

Unlabeled vertex *v_u_* and labeled seed point $V_{m},V_{U}\cap V_{M}=\phi ,V_{U}\cup V_{M}=V$ . The sum of probability of each vertex arriving at each tag is 1. After calculating the probability of any vertex *v_i_* arriving at each kind of tag, vertex *v_i_* is classified according to the maximum probability criterion.

### 2.3 Feature Extraction of Lung CT Images Based on Machine Learning

The texture feature and gray feature of the image can well express the characteristics of the target region. In this paper, the texture feature based on the local directional tri-value pattern and the gray feature based on the gray histogram are used to extract the features from the lung CT images. Among them, texture features are 39 dimensions, gray features are 12 dimensions, a total of 51 dimensions.

#### 2.3.1 Pulmonary CT Image Texture Feature Extraction Based on Volume Local Directional Trinary Mode

The volume local directional tri-value model is a method to calculate the three-dimensional local texture features of pulmonary nodules based on the texture orientation and variation in the time dimension. The idea of extracting the texture features of pulmonary nodules based on the three-value model of body local direction is as follows: First, pulmonary nodules were sequenced. Secondly, the adjacent sections of pulmonary nodules were extracted one by one. The normal function is used to calculate the pixel value of the local mode. Finally, the -1, 0 and +1 in the 13 directions of the central pixel were counted according to the obtained volume local three-value model to form the dimensional feature vectors. Add the eigenvectors of all local modes and then normalized to form the final texture feature vectors of pulmonary nodules.

Where, the center pixel is the mean value of all pixel values in the local mode, and the offset pixel is the variance of all pixel values in the neighborhood in the local mode. The calculation formula for the relationship between the center pixel and the neighborhood pixel is shown in Formula 9.


(9) $$\vec{f}\left(g_{p},\mu ,\sigma ,k\right)=\{\begin{array}{c} +1,g_{p}>\mu +k\sigma \\ 0,|g_{p}-\mu |\leq k\sigma \\ -1,g_{p}<\mu -k\sigma \end{array}$$

Where, *μ* represents the center pixel obtained by calculating the mean value according to the center pixel value and 26 neighborhood pixel values; *g_p_* represents neighborhood pixels; σ represents the fixed threshold value obtained from the mean square error calculation based on the center pixel value and 26 neighborhood pixel value, and *k* is the threshold coefficient;

The calculation formula of local adaptive threshold σ is as follows:


(10) $$\sigma =\sqrt{\frac{1}{N-1}\sum_{i=1}^{N-1}{\left(x_{i}-\mu \right)}^{2}}$$

Where, *x_i_* represents the pixel value of *i*, *μ* represents the mean value of pixels in the local mode, and *N* represents the number of pixels in the local mode, with a value of 27.

In the slice image of pulmonary nodules, the pixel distribution conforms to the characteristics of normal function, Therefore, according to the distribution table of normal function, the value calculation formula is as follows:


(11) $$S_{1}+S_{2}=\int_{-\infty }^{k}\frac{1}{\sqrt{2\pi }}\exp\left\{-\frac{x^{2}}{2}\right\}dx=\Phi (k)$$

The experimental results show that when *k* = 1, the texture features based on the tri-value pattern of body local direction can obtain the most accurate results of nodular classification.

Statistically calculate the trivalues -1, 0, + 1 in 13 directions to form a feature vector of 13 and 3 dimensions, and connect the sum of the trivalues in all directions to form a feature vector, and the calculation formula is shown in 12:


(12) $$H(VLDTP)=\sum_{i=2}^{N_{1}-1}\sum_{j=2}^{N_{2}-1}\sum_{k=2}^{N_{3}-1}f\left(LTP\left(i,j,k\right),\theta \right)$$

Where, *N*_1_ × *N*_2_× *N*_3_ represents the size of the 3D image, the *LTP* local ternary mode, (*i,j,k*) represents the pixels in the *i* row, *j* column and *k* layer of the image, and *θ* shows all directions of (*i,j,k*). The normalized eigenvector is shown in formula 13:


(13) $$H(NVLDTP)=\frac{H(VLDTP)}{\left(N_{1}-2)(N_{2}-2)(N_{3}-2\right)}$$

**Figure 4:**
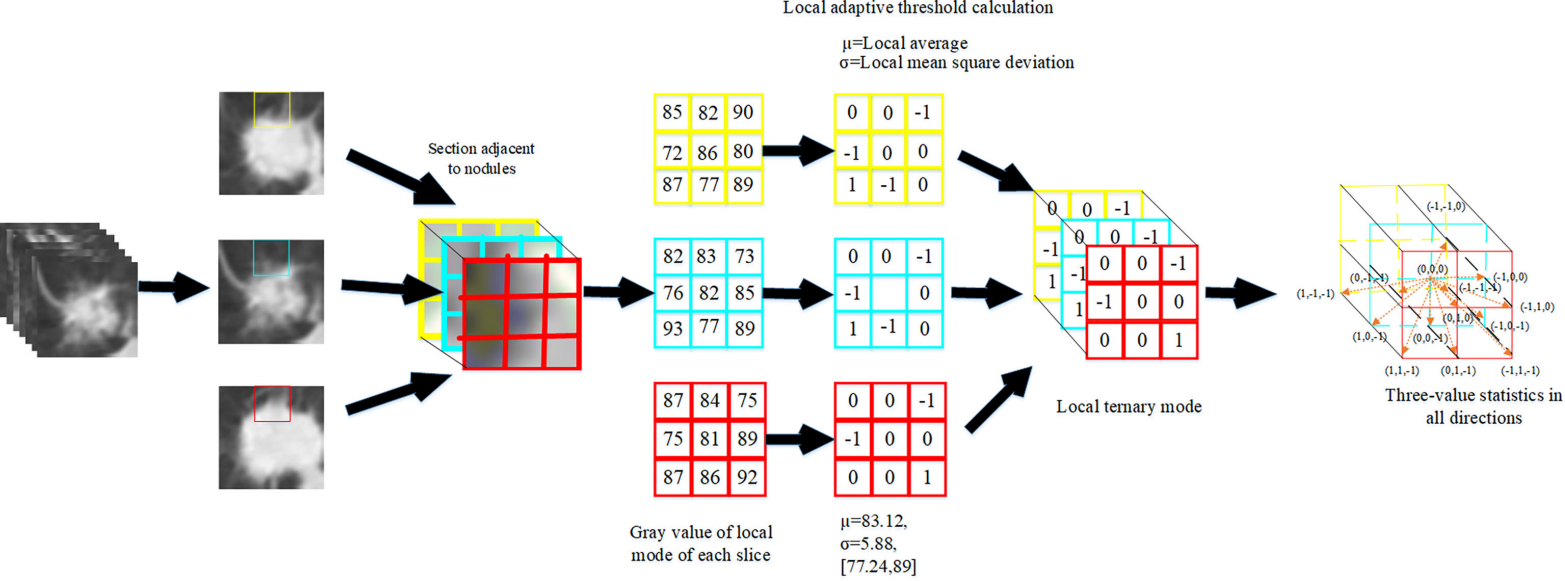
Extraction process of texture features of pulmonary nodules.

The extraction process of pulmonary nodules texture features is shown in **Figure 4**.

#### 2.3.2 Gray Scale Feature Extraction of Lung CT Images Based on Gray Histogram

As the most basic statistical feature of images, gray histogram is a function of gray level distribution. The gray histogram can be used to conduct statistics on the gray value of pixels in the image, reflecting the occurrence frequency of certain gray value pixels in the image (24). Based on the features of gray histogram, 12 gray features including mean value, median, standard deviation, skewness, kurtosis, minimum value, maximum value, entropy, energy, range, mean absolute deviation and root mean square of the pixel value were extracted from the pulmonary nodules section, and finally a dimensional feature vector was formed.

The calculation formula of gray histogram is as follows:


(14) $$p(r_{k})=\frac{n_{k}}{n}$$

Where, *r_k_* is the gray level of pixels, *n_k_* is the number of pixels with gray level *r_k_*, and *n* represents the total number of pixels in the image.

### 2.4 Pulmonary Nodule Classification Based on VGG16 Model

The VGG16 network was verified by the Visual Geometry Group of the Department of Engineering Sciences at the University of Oxford in 2014 at the ImageNet Challenge, and the results of the competition showed that the network classification performance was greatly improved by using 3 × 3 convolutional filters and increasing the depth to 16-19 weight layers (25). Zhang Haitao (26) and Yifei Chen (27) used VGG16 convolutional neural network to study the classification of medical image data, which indicated that it was feasible to use VGG16 network to complete pulmonary nodules classification.

**Figure 5:**
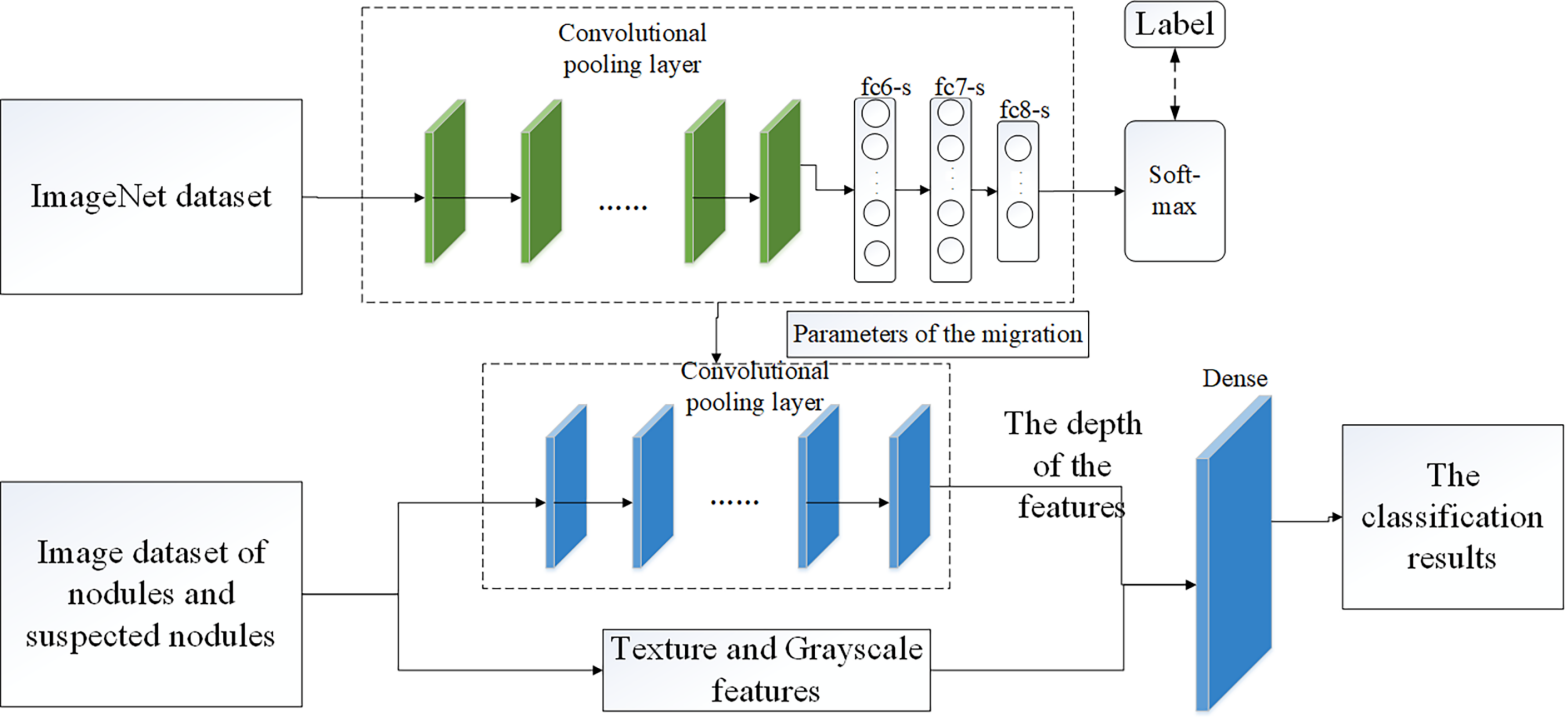
Classification model of pulmonary nodules.

We did not make any changes to the VGG16 network structure. In the classification of pulmonary nodules using VGG16 network, we processed 1000 data, including 500 nodular images and 500 suspected nodular images, and divided them into training sets and test sets in an 8:2 ratio. Before this, we adopt the method of parameter migration, and take the weight parameters trained by ImageNet data set as the weight parameters of VGG16 network model. Then, interpretable texture features and grayscale features were fused with depth features in series in the VGG16 network to complete the final nodule classification. **Figure 5** shows the classification model of pulmonary nodules.

### 2.5 Evaluation Indicators

#### 2.5.1 Evaluation of Lung Parenchyma Segmentation

In this paper, Intersection of Union (IOU) and false positive were used to verify the effectiveness of the proposed method. Among them:

*TP*: Indicates that it is actually a positive sample and is judged to be a positive sample at the same time, that is, the true positive count;

*FP*: Indicates that it is actually a negative sample and is judged to be a positive sample, that is. a false positive count;

*FN*: Indicates that it is actually a positive sample and is judged as a negative sample, that is, a false negative count;

*TN*: Indicates that it is actually a negative sample and is judged to be a negative sample, i.e., a true negative count.

Intersection of Union (IOU) represents the similarity between the predicted area and the real area in a group of images. The formula is shown in Equation (15).


(15) $$IOU=\frac{TP}{FP+TP+FN}$$

The false positive rate represents the probability of the actual negative samples being misjudged as positive samples. The smaller the false positive rate, the better. The formula is shown in Equation (16).


(16) $$FPR=\frac{FP}{FP+TN}$$

#### 2.5.2 Evaluation Index of Pulmonary Nodule Classification

Six evaluation indexes, accuracy, sensitivity, specificity, accuracy, F1 score and Standard error were used to verify the experimental method. The formulas are shown in Equations (17), (18), (19), (20) (21) and (22). The confusion matrix is shown in **Table 2**.


(17) $$Accuracy=\frac{\left(TP+TN\right)}{\left(TP+TN+FP+FN\right)}$$

(18) $$Sensitivity=\frac{TP}{\left(TP+FN\right)}$$

(19) $$Specificity=\frac{FP}{\left(FP+TN\right)}$$

(20) $$Precision=\frac{TP}{\left(TP+FP\right)}$$

(21) $$F_{1}=2\times \frac{Precision\times Sensitivity}{Precision+Sensitivity}$$

(22) $$Standard\;error=\sqrt{\frac{1}{n-1}\sum_{i=1}^{n}v_{i}^{2}}$$

**Table 2:** Confusion matrix.

		The real value	
		1	0
Predictive value	1	TP	FP
	0	FN	TN

## 3 Results

**Table 3** shows the results of lung parenchyma region obtained from five lung parenchyma images using three segmentation methods. **Table 4** shows the comparison of IOU and false positive rate of lung parenchyma region obtained from five lung parenchyma images using three segmentation methods. **Table 5** shows the nodular classification results of BP neural network based on features obtained by different methods. Two nodule texture features were obtained based on volume local triadic direction and three-dimensional gray co-occurrence matrix, which verified that the feature factors obtained by different methods had an impact on the classification of nodules. At the same time, we input the texture features obtained by both methods into BP neural network in series with gray features, respectively, and we prove that multiple features can affect nodule classification. **Table 6** shows the results of nodule classification under different classifiers and different nodule characteristics.

**Table 3:** Comparison of lung parenchyma segmentation results.

	The original image	Interactive random walk algorithm for lung parenchyma segmentation	Automatic random walk algorithm for lung parenchyma segmentation based on original weight function	Automatic random walk algorithm for lung parenchyma segmentation based on improved weight function
example 1	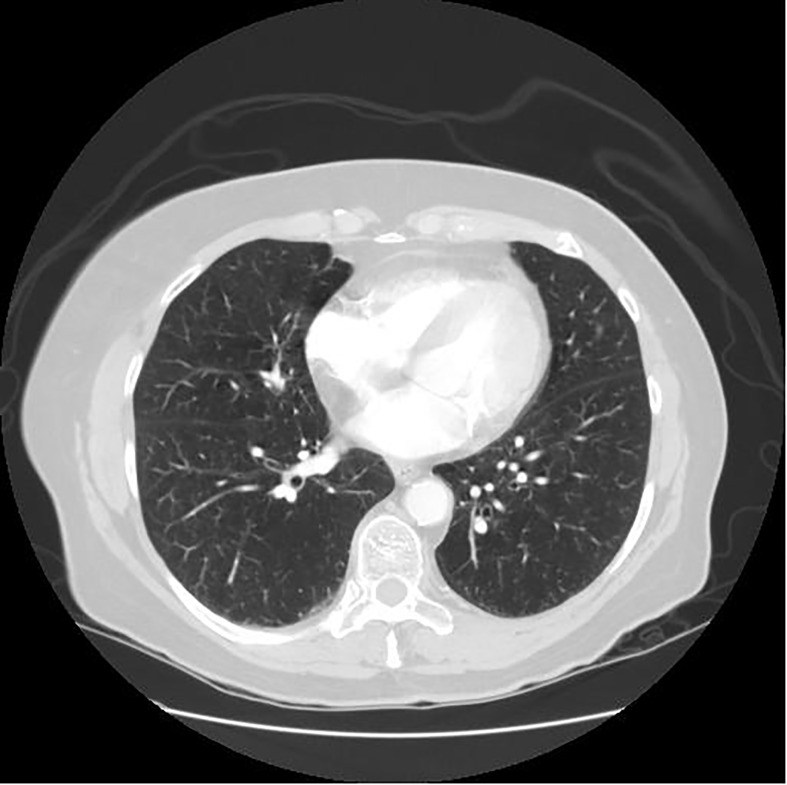	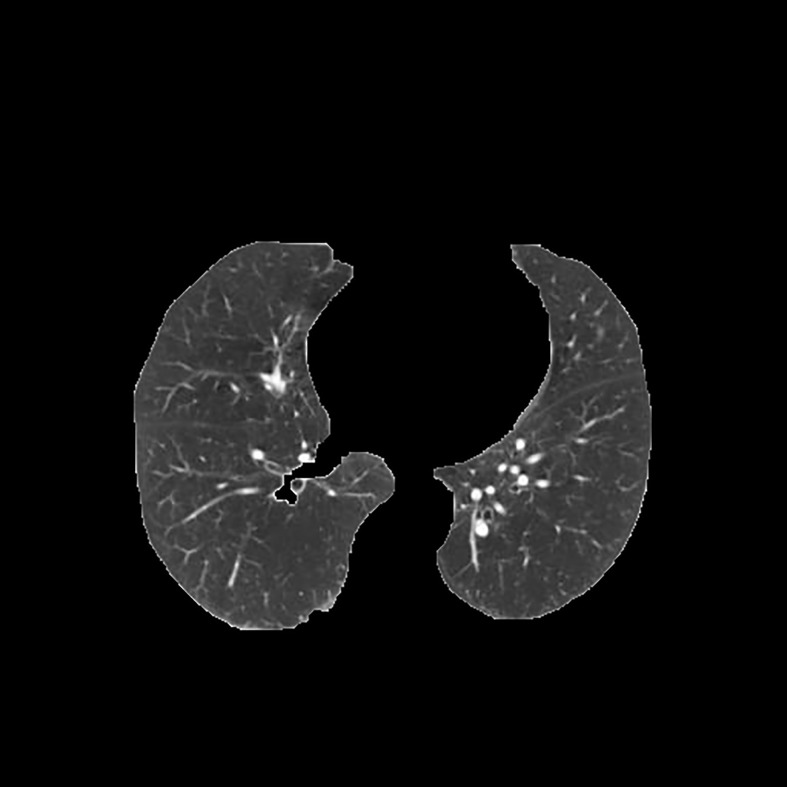	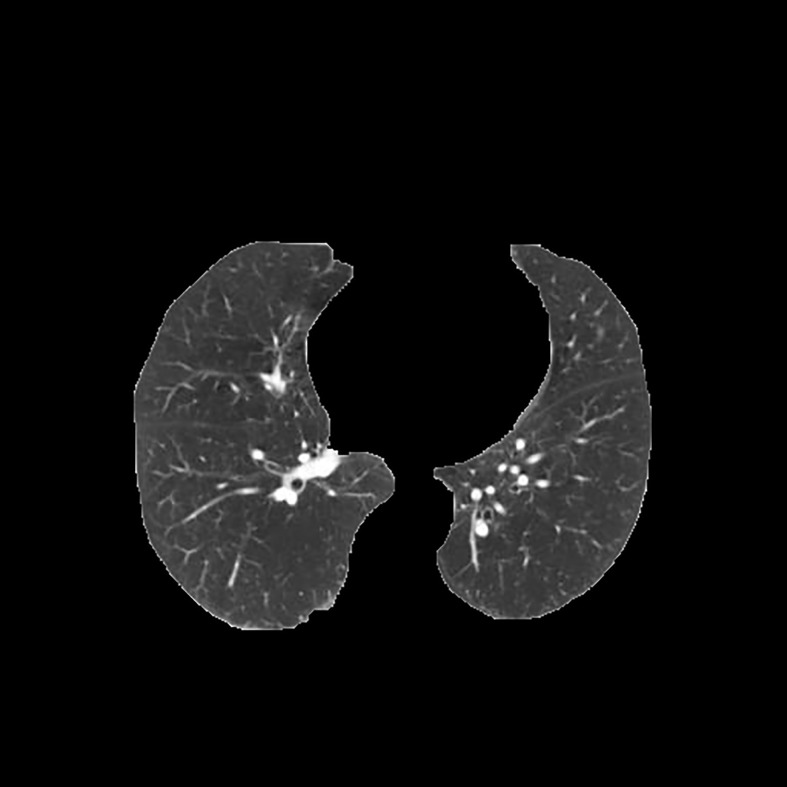	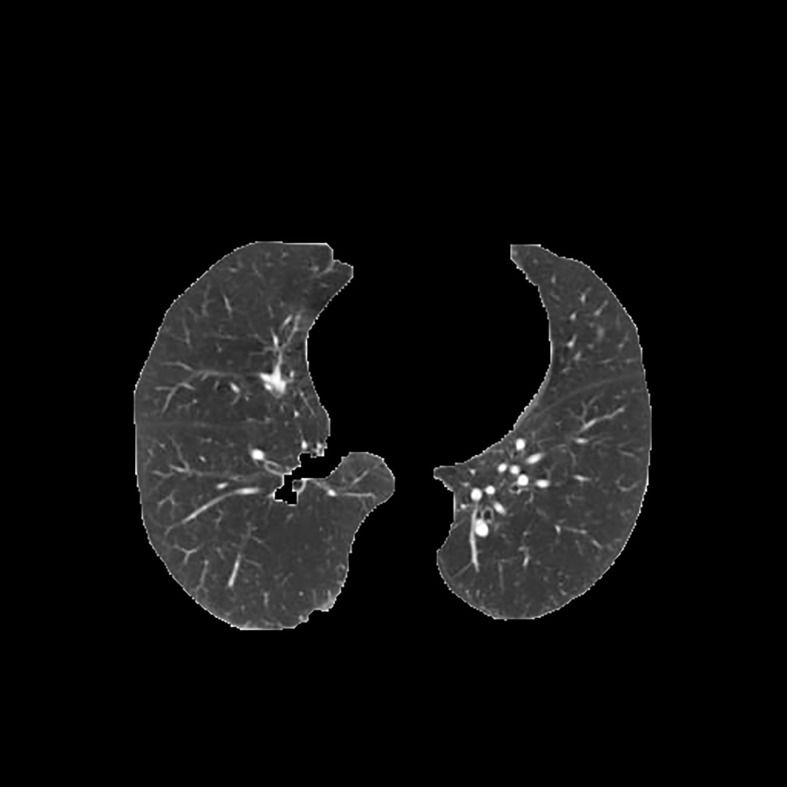
example 2	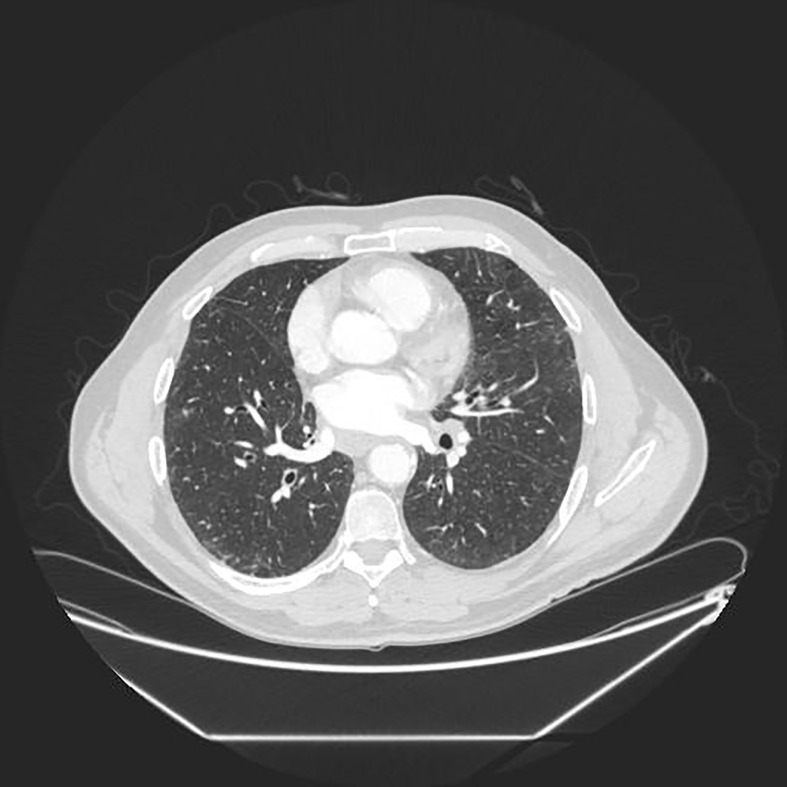	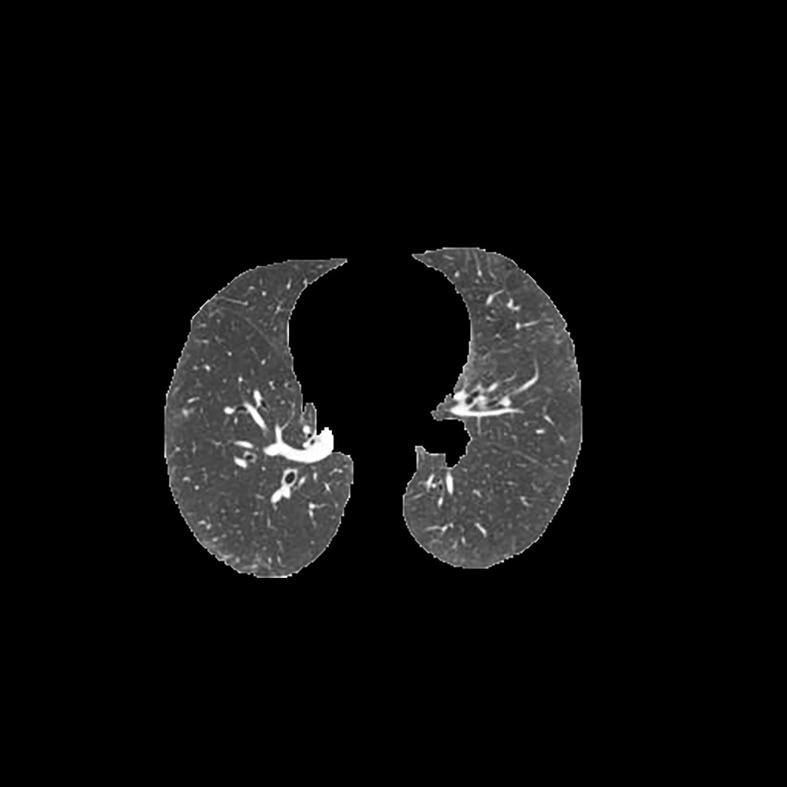	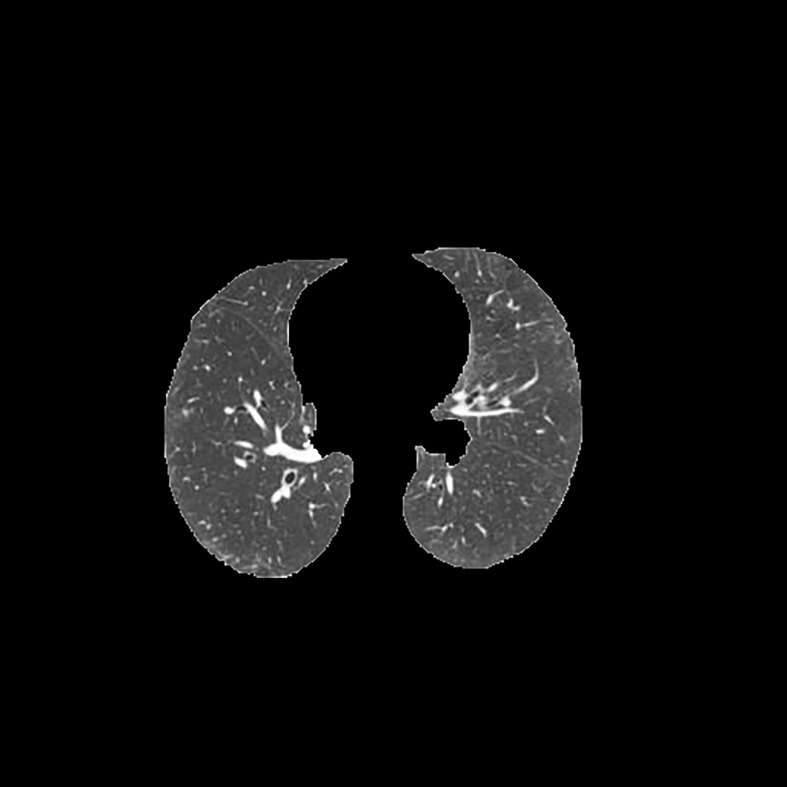	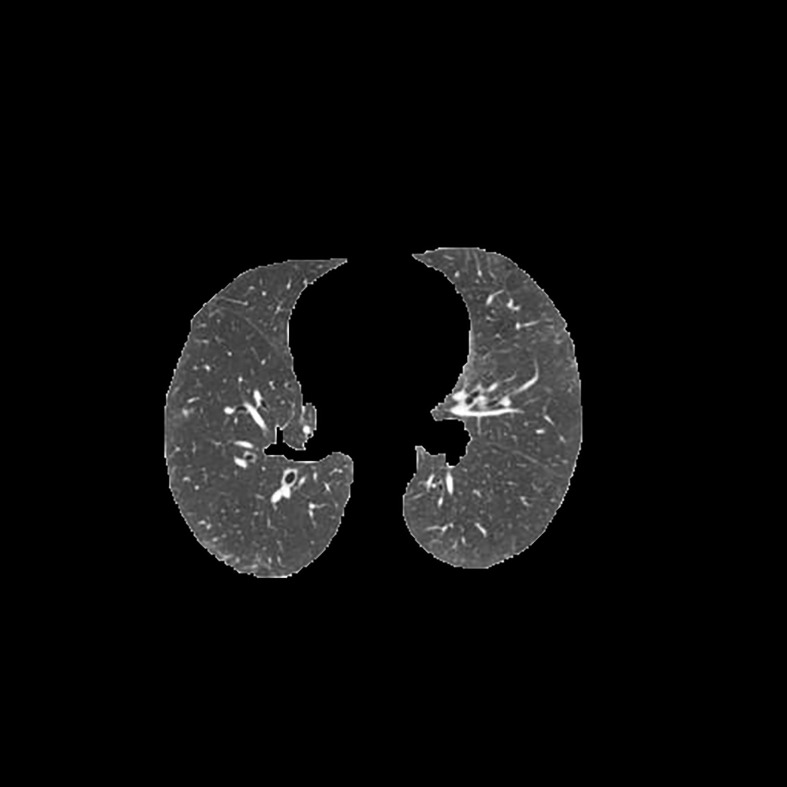
example 3	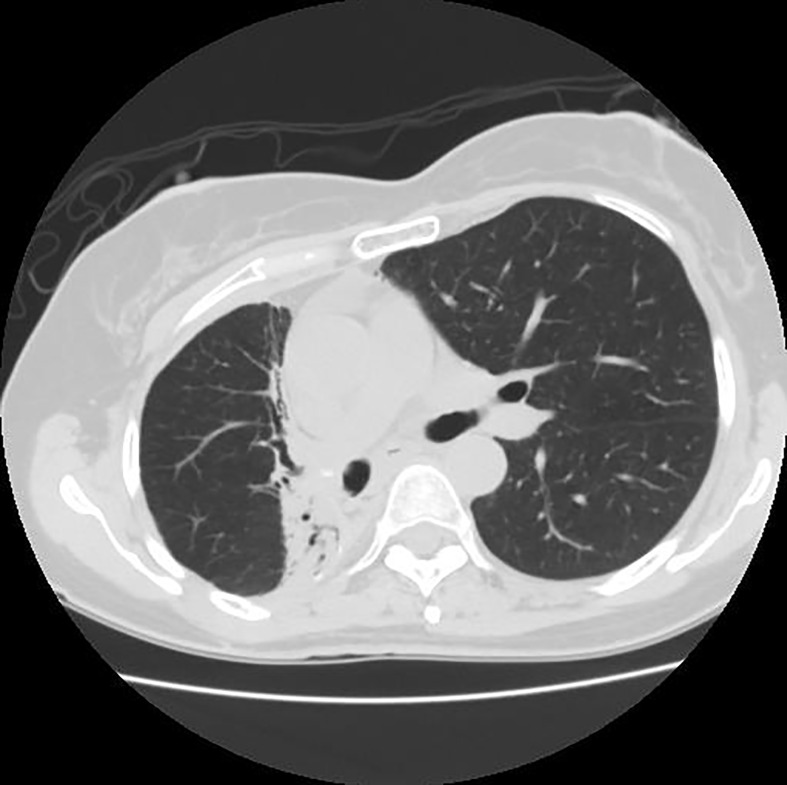	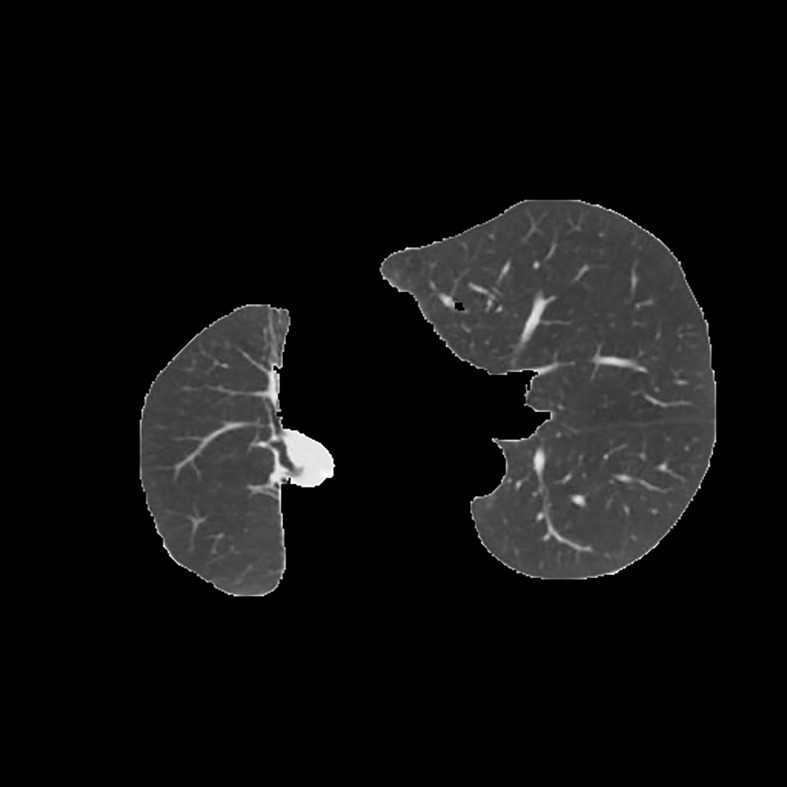	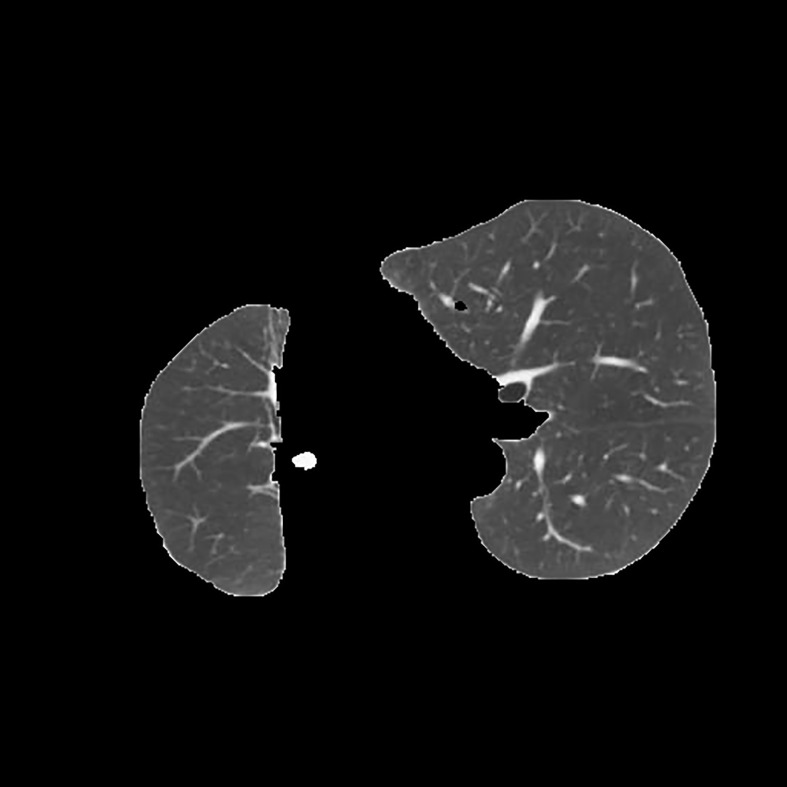	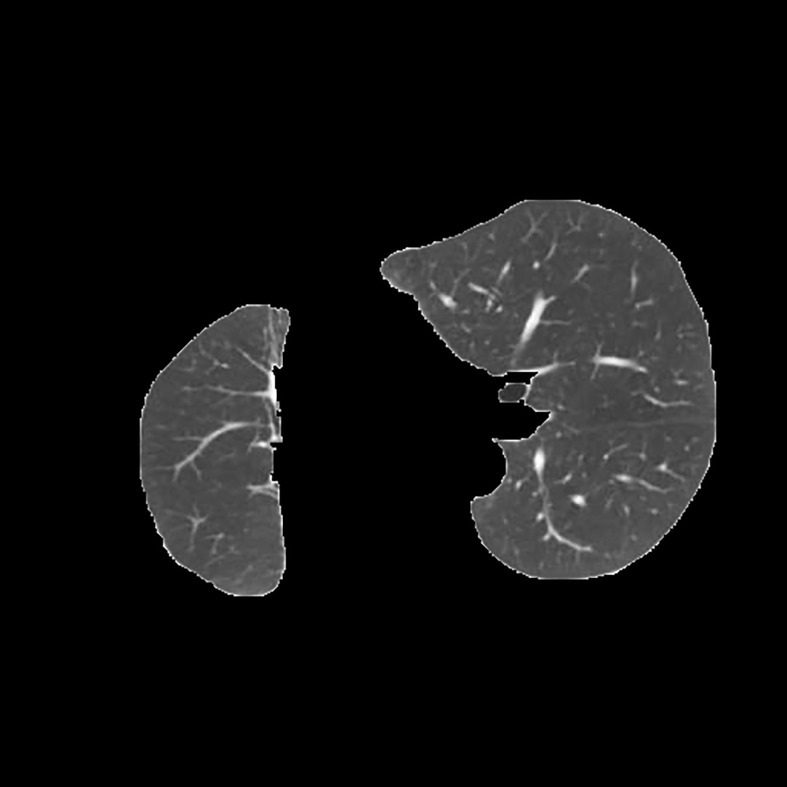
example 4	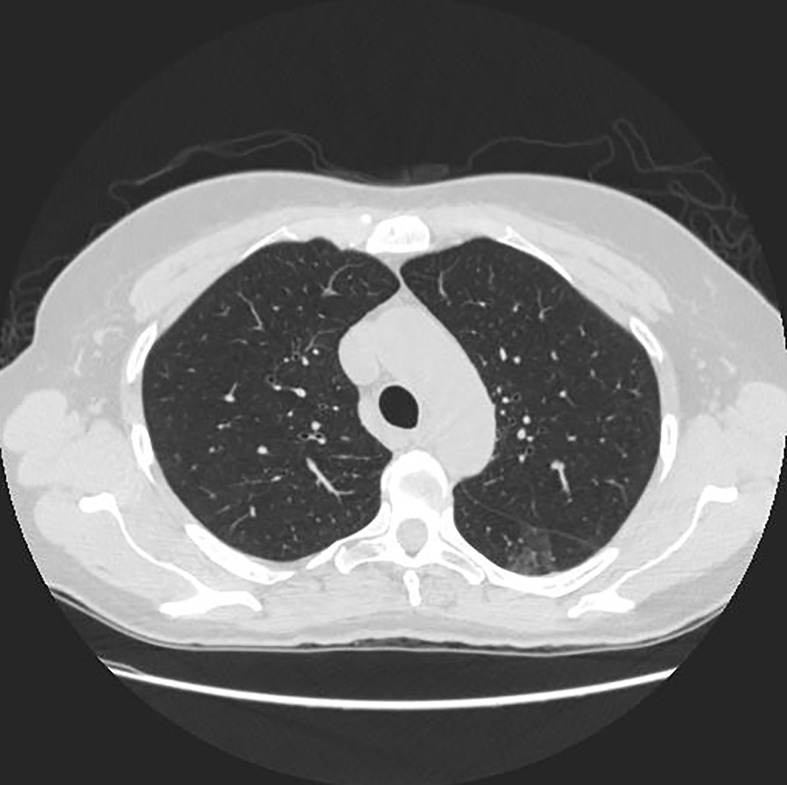	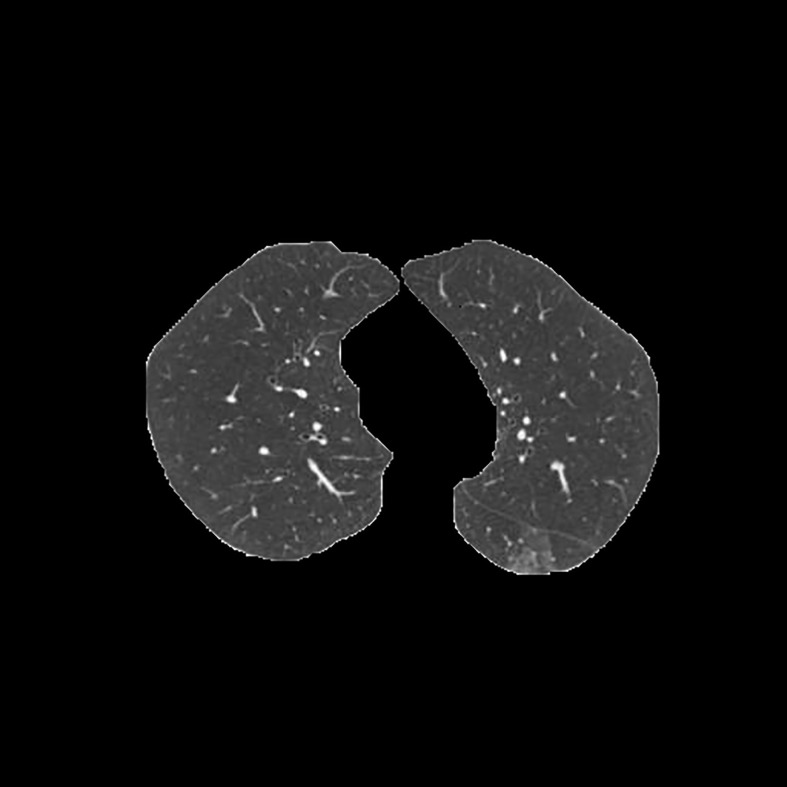	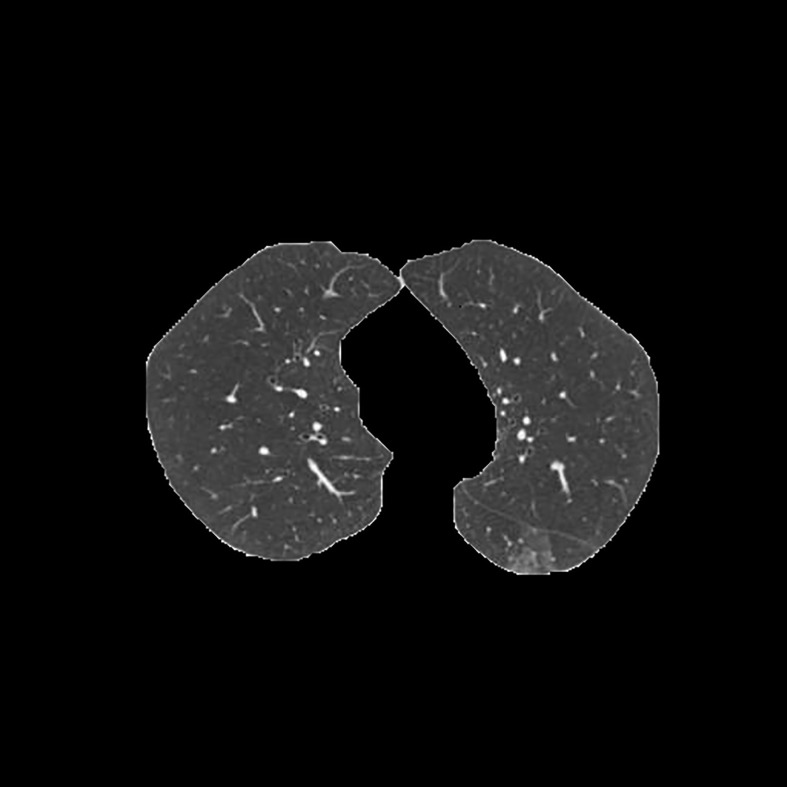	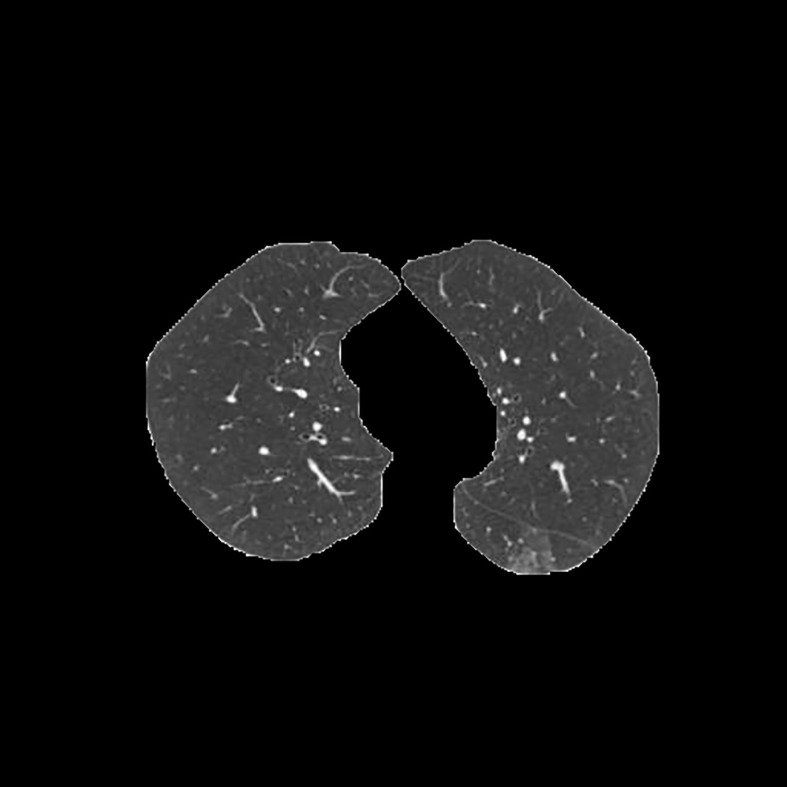
example 5	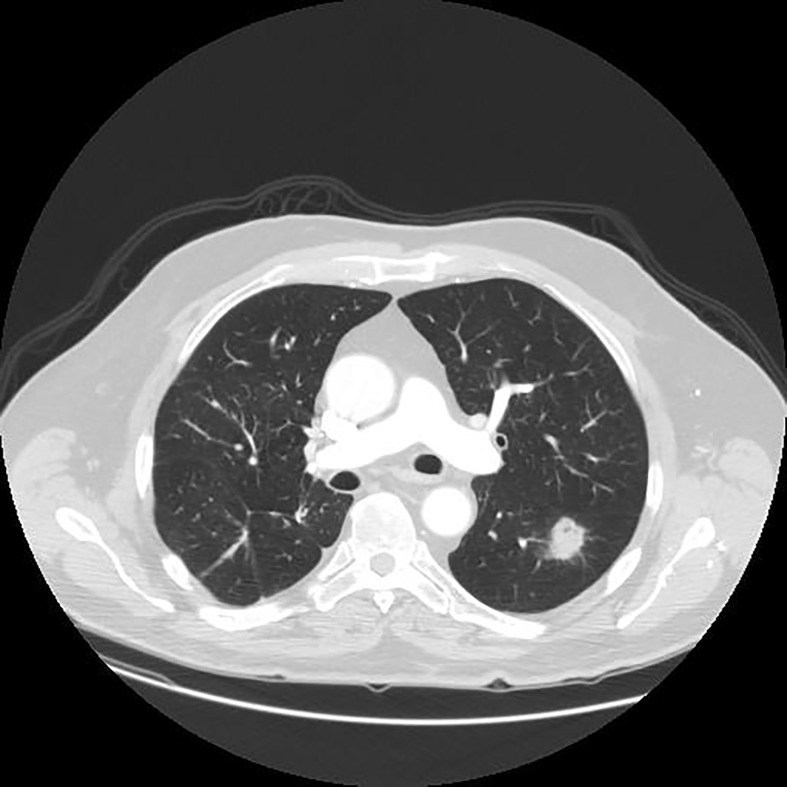	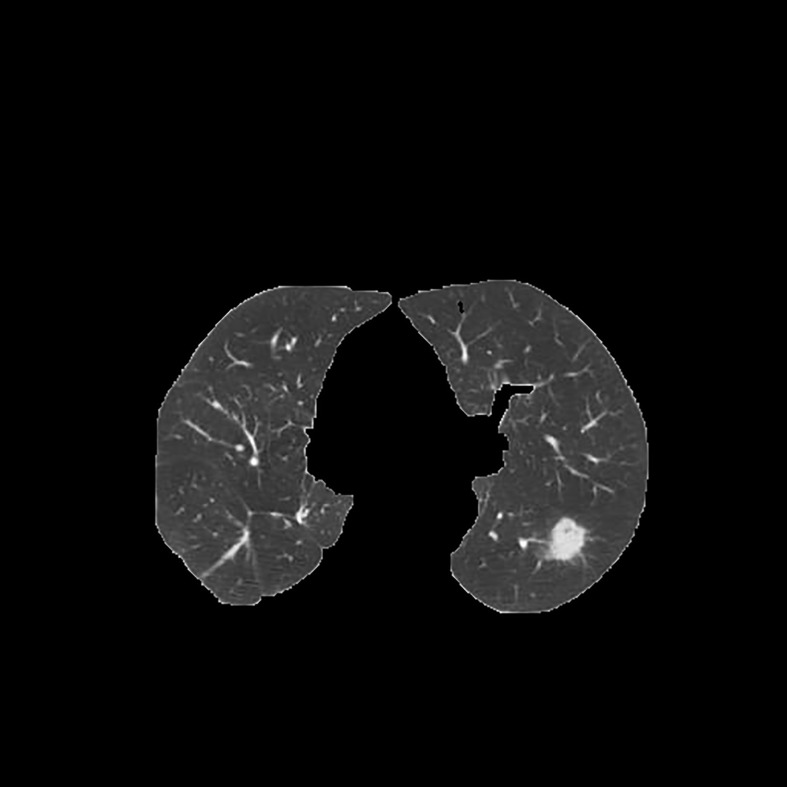	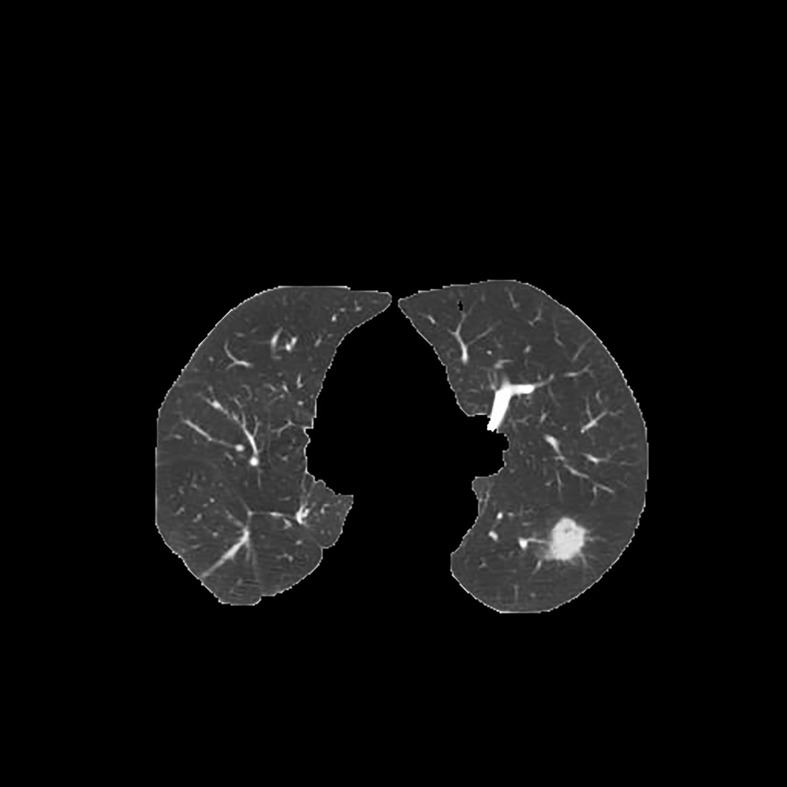	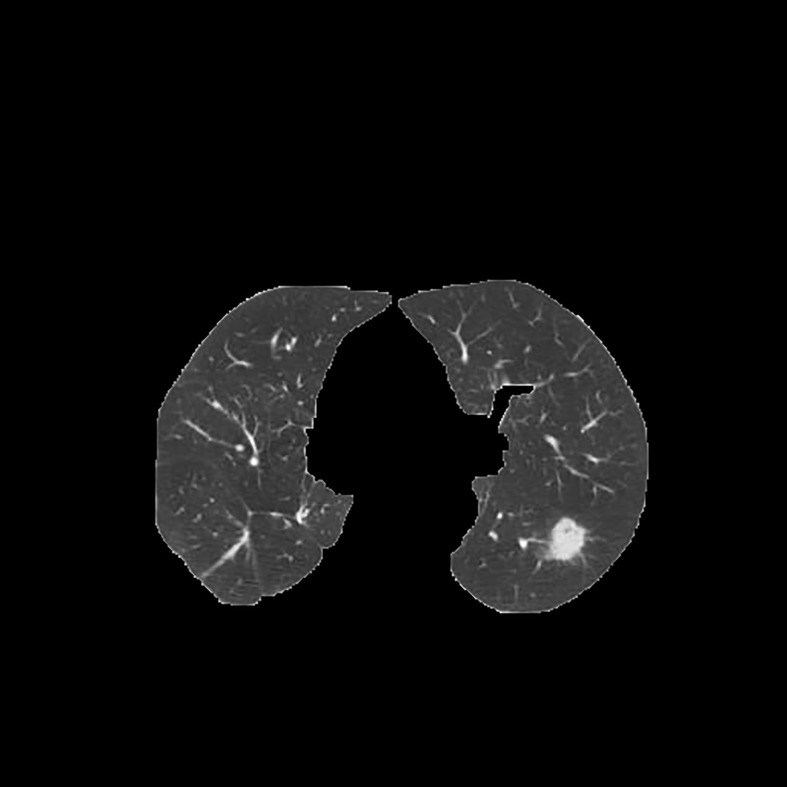

**Table 4:** Segmentation and contrast data of lung parenchyma.

	Interactive random walk algorithm for lung parenchyma segmentation		Automatic random walk algorithm for lung parenchyma segmentation based on original weight function		Automatic random walk algorithm for lung parenchyma segmentation based on improved weight function	
	IOU	FPR	IOU	FPR	IOU	FPR
**Figure 1**	0.977257	0.021624	0.967491	0.031845	0.978620	0.020667
**Figure 2**	0.945236	0.054284	0.949879	0.049638	0.960230	0.039038
**Figure 3**	0.948815	0.045924	0.969174	0.029350	0.976540	0.022634
**Figure 4**	0.988972	0.009077	0.988602	0.010148	0.990128	0.008088
**Figure 5**	0.984976	0.013224	0.977358	0.021071	0.986518	0.011872

IOU, Intersection of Union, FPR, false positive rate.

**Table 5:** Comparison of the classification results of pulmonary nodules by BP neural network.

Feature extraction method	Accuracy	Sensitivity	Specificity	Precision	F1 Score
VLDTP	0.875	0.8889	0.8614	0.8627	0.8756
VLDTP+ Gray histogram	0.88	0.89	0.87	0.87.25	0.8812
3D-GLCM	0.77	0.8333	0.6957	0.7964	0.7627
3D-GLCM+ Gray histogram	0.86	0.8687	0.8515	0.8515	0.86

3D-GLCM, 3-Dimensional Gray-Level Co-Occurrence Matrix, VLDTP, Volume Local Direction Ternary Pattern.

**Table 6:** Comparison of nodular classification results by different methods.

Classifier	Accuracy	Sensitivity	Specificity	Precision	F1 Score	Standard error
BP neural network	0.88	0.89	0.87	0.8725	0.8812	17.03
Single image feature VGG16 network	0.93	0.9327	0.9271	0.9327	0.9327	9.90
Multi-feature VGG16 network	0.975	0.9681	0.9811	0.9785	0.9733	3.61

## 4 Discussion

In this study, we proposed an automatic random walk method for lung parenchyma segmentation, and proposed that the texture features extracted based on local ternary direction and gray features extracted based on gray histogram were fused with depth features in VGG16 network in series to complete nodule classification. Our results show that on the basis of automatic seed point acquisition of random walk, adding the shortest distance between sets can improve the accuracy of lung parenchyma segmentation, and adding interpretable nodule features in VGG16 network can effectively improve the accuracy of nodule classification.

In pulmonary parenchyma segmentation, the manual interactive random walk segmentation method usually needs a lot of time to determine the seed point of the target region before segmentation can be completed. And in this process will be affected by human factors, the determined seed point has randomness. Therefore, we propose a random walk segmentation method for automatic seed point acquisition. When we add the shortest distance between sets to the weight function calculation, our segmentation effect is better, and the problem of incomplete segmentation between two lungs is solved. This may be because the distance between pixel vertex and the nearest target seed point can be calculated in the original random walk weight function, which can more accurately complete the classification of pixel points, so as to achieve accurate segmentation. In early lung parenchyma segmentation based on machine learning, Guo, Y. R. et al. integrated texture information into random walk weight function (28). Although effective in segmentation, it was limited to manual interactive random walk segmentation. Wang, G. L. et al. added Euclidean distance to the weight function (29). However, as far as we know, there is no research report that calculates the shortest distance between sets in the weight function. Our results and the study of Wang, G. L. et al. show that calculating the distance between pixels is conducive to improving the accuracy of random walk segmentation.

In nodular classification, the fusion of explicable features and depth features can improve the classification accuracy, possibly because the explicable features extracted by us add more identifiable data to the depth features. For nodular classification based on deep learning, most of the input data are single image data (30) or depth features are input into traditional classifier (31, 32). At present, there are few studies that take interpretable features and image data as the input of neural network, and the results of different interpretable features input into neural network are different to some extent. Therefore, our experiment verifies the classification effect of the fusion of texture feature based on Volume Local Direction Ternary Pattern and gray feature based on Gray Histogram extraction and depth feature in VGG16 network, and the experiment shows that the method is feasible.

There are still some limitations in our study. Firstly, the automatic random walk segmentation algorithm proposed by us is only applied to lung parenchyma segmentation, and the effectiveness of this method in segmentation of other organs has not been verified. Secondly, in the nodular classification, the weight parameters of the VGG16 model we used were obtained by ImageNet data set training instead of a large number of nodular data sets, which would cause some errors when we used this model for pulmonary nodular training.

In conclusion, the automatic random walk segmentation algorithm proposed by us is effective in lung parenchyma segmentation. In VGG16 network, the depth features are fused with texture features extracted based on Volume Local Direction Ternary Pattern (VLDTP) and gray features extracted based on Gray Histogram in series mode, which can improve the accuracy of nodular classification. However, In order to further improve the model reliability, a larger nodule dataset should be used to replace the ImageNet dataset to train the weight parameters of the model.

## Data Availability Statement

Publicly available datasets were analyzed in this study. This data can be found here: https://wiki.cancerimagingarchive.net/display/Public/LIDC-IDRI.

## Ethics Statement

Written informed consent was not obtained from the individual(s) for the publication of any potentially identifiable images or data included in this article.

## Author Contributions

Conceptualization, YZ and LM. Methodology, YZ and LM. Software, YZ and LM. Validation, LM. Formal analysis, YZ. Investigation, LM. Resources, YZ. Data curation, LM. Writing—original draft preparation, LM. Writing—review and editing, YZ. Visualization, YZ and LM. Supervision, YZ. project administration, YZ. Funding acquisition, YZ. All authors have read and agreed to the published version of the manuscript.

## Funding

Heilongjiang Provincial Natural Science Foundation Joint Guide Project in 2021, No. LH2021F036.

## Conflict of Interest

The authors declare that the research was conducted in the absence of any commercial or financial relationships that could be construed as a potential conflict of interest.

## Publisher’s Note

All claims expressed in this article are solely those of the authors and do not necessarily represent those of their affiliated organizations, or those of the publisher, the editors and the reviewers. Any product that may be evaluated in this article, or claim that may be made by its manufacturer, is not guaranteed or endorsed by the publisher.
